# ETS1-activated SNHG10 exerts oncogenic functions in glioma via targeting miR-532-3p/FBXL19 axis

**DOI:** 10.1186/s12935-020-01649-2

**Published:** 2020-12-09

**Authors:** Lide Jin, Shengquan Huang, Congjin Guan, Shun Chang

**Affiliations:** The First People’s Hospital of Yunnan Province, The Affiliated Hospital of Kunming University of Science and Technology, No.157 Jinbi Road, Kunming, 650032 Yunnan China

**Keywords:** SNHG10, miR-532-3p, FBXL19, Glioma, ETS1

## Abstract

**Background:**

In past few years, long non-coding RNAs (lncRNAs) have been reported to play regulatory roles during cancer progression. LncRNA SNHG10 has been explored in several sorts of cancers. However, its detailed role and mechanism are still not well understood in glioma.

**Methods:**

Expression levels of genes were evaluated by RT-qPCR. EdU, TUNEL, sphere formation, wound healing and transwell assays appraised the effect of SNHG10 on glioma cellular processes. The interaction between molecules was examined by ChIP, RIP, RNA pull down and luciferase reporter assays.

**Results:**

High level of SNHG10 was detected in glioma cells. Functional assay confirmed that SNHG10 promoted the proliferation, migration, invasion and stemness of glioma cells. Moreover, miR-532-3p was validated to bind with SNHG10 and expressed at a low level in glioma cells. Importantly, miR-532-3p exerted inhibitory functions in glioma. Furthermore, it was found that FBXL19 targeted by miR-532-3p facilitated cell growth and stemness in glioma, and that SNHG10 worked in glioma by increasing FBXL19 expression through sequestering miR-532-3p. More importantly, ETS1 promoted the transcription of SNHG10 and it mediated contribution to the malignant behaviors of glioma cells by SNHG10/miR-532-3p/FBXL19 signaling.

**Conclusion:**

SNHG10 was transcriptionally activated by ETS1 and played an oncogenic role in glioma by sponging miR-532-3p and up-regulating FBXL19. 
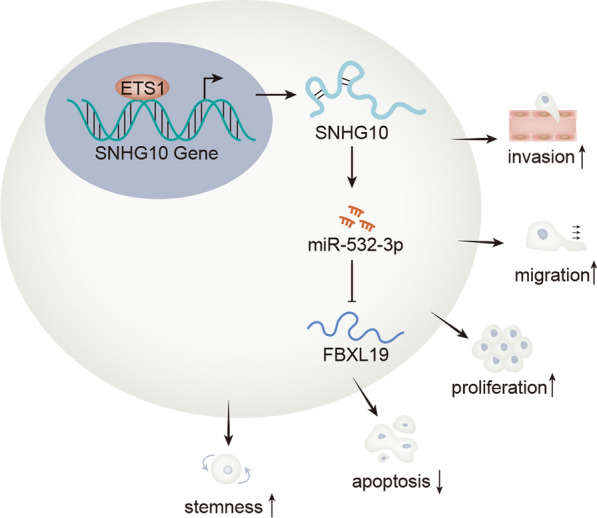

## Background

Glioma is considered as one of the most common tumors occurring in human brain and occupies around 80% of total brain tumors [1, 2]. According to recent reports, glioma could be classified into four grades according to malignancy and lesion location [3]. The invasive characteristic of high-grade glioma determines that there is no distinctive boundary between glioma and normal brain tissue [4, 5]. Moreover, the current radiotherapy and chemotherapy had limited therapeutic effects on glioma [6, 7]. Hence, it is necessary to find more efficient biomarkers for improving the treatment of glioma patients.

In recent years, attentions have been increasingly attached into target therapy due to the advance of scientific technology and biology. Long non-coding RNAs (lncRNAs) are identified to play crucial roles in the development of cancers [8]. Numerous lncRNAs are demonstrated to have potential values in treating tumors [9]. LncRNA PLAC2 reduced the expression of RPL36 and repressed cell cycle progression in glioma by targeting STAT1 [10]. LncRNA H19 was up-regulated in glioma cells and predicted poor survival rates [11]. LncRNA GHET1 was an oncogene since it aggravated the malignant biological behaviors in glioma [12]. SNHG10 is a novel lncRNA which has not been explored in glioma.

Increasing studies supported that competing endogenous RNA (ceRNA) regulatory network exerted essential functions in modulating the development of glioma [13, 14]. In ceRNA network, lncRNAs increase mRNA expression to regulate cancer progression by sponging miRNAs. For example, lncRNA CASC2 functioned as the sponge of miR-181a to regulate glioma progression and the resistance of glioma cells to TMZ by targeting PTEN pathway [15]. LncRNA FOXD2-AS1 expedited glioma cell growth and drug resistance via sponging miRNA-98-5p to up-regulate CPEB4 [16]. In this study, we aimed to investigate whether SNHG10 mediated ceRNA network in glioma.

MiRNAs are also important regulators in the development of cancers including glioma [17]. For instance, miR-215 exerted oncogenic functions in high-grade glioma via modulating RB1 expression [18]. MiR-133b suppressed cell proliferation and invasion in glioma through targeting Sirt1 [19]. MiR-532-3p has been unveiled to have opposite influences in different cancer types [20, 21], but it has not been researched in glioma.

The main task of our study was to investigate the function and molecular mechanism of SNHG10 in glioma. As a result, ETS1/SNHG10/miR-532-3p/FBXL19 axis was discovered, which might provide a helpful theoretic basis for the exploration of new targets for glioma therapy.

## Methods

### Cell culture

Human glioma cell lines (U138, LN-229, U251, T98G and A-172) and normal cell line (NHA) were all available from the ATCC cell bank (Manassas, VA) for cell culture under 5% CO_2_ and 37 ℃. DMEM was procured from Gibco (Grand Island, NY), along with the 10% FBS and 1% pen/Strep solution in the culture medium.

### Extraction of total RNA and RT-qPCR

The extraction of total RNAs was performed with the TriZol reagent as per the protocol (Invitrogen, Carlsbad, CA), and then the obtained RNAs were subjected to reverse-transcription. Using SYBR^®^ Premix Ex Taq™ II, the quantitative PCR was performed to monitor the gene expression as guided by the supplier (Takara Bio Inc. Shiga, Japan). The data relative to GAPDH or U6 were processed with the 2^−ΔΔCt^ method. Primer sequences were all shown in Table 1.

**Table 1:** The sequences of primers used in RT-qPCR

Primers	Sequences (5′–3′)
SNHG10-F (forward)	CAAGCCTCATCAGGCCCATT
SNHG10-R (Reverse)	AGTCCACTGGTCCTGCTCTT
OCT4-F	CCTTCGCAAGCCCTCATTTC
OCT4-R	TAGCCAGGTCCGAGGATCAA
Nanog-F	CCCCTAATTTGTTGGTTGTGCT
Nanog-R	GCTAATTTCCTTCTCCACCCCA
SOX2-F	TGGACAGTTACGCGCACAT
SOX2-R	CTCGGACTTGACCACCGAAC
GAPDH-F	GGAGCGAGATCCCTCCAAAAT
GAPDH-R	GGCTGTTGTCATACTTCTCATGG
U6-F	CTCGCTTCGGCAGCACA
U6-R	AACGCTTCACGAATTTGCGT
FBXL19-F	AACTCATCTCCAGCCCTCCTT
FBXL19-R	CCATCCATGGGGCTAAGGG
NPTN-F	GGAAGACCCTGGCGAGTATG
NPTN-R	TCCAGCTGGTTCATCATCGT
SRRM2-F	ATGAGACACCGCTCCTCCA
SRRM2-R	GCTGCCAAGGTTCAAAGGAG
ETS1-F	TGTAGCGATGTAAGTGTCGATG
ETS1-R	GATGTGCCAGCATCAGCTAC
miR-2278-F	ACGAGGAGAGCAGTGTGTGTT
miR-532-3p-F	ATAATCCTCCCACACCCAAGG
miRNA-R (universal)	CTCAACTGGTGTCGTGGA

### Plasmid transfection

The designed shRNAs and relative negative control (NC) (sh-NC) for SNHG10, FBXL19 and ETS1 were procured from Genepharma Company (Shanghai, China) to transfect U138 and U251 cells employing Lipofectamine 2000 (Invitrogen). Besides, the pcDNA3.1/SNHG10, pcDNA3.1/FBXL19, and pcDNA3.1/ETS1 were constructed by inserting corresponding cDNA of indicated genes into pcDNA3.1 vectors (Invitrogen), with the empty pcDNA3.1 as NC. Also, the miR-532-3p mimics/inhibitor and NC mimics/inhibitor, were all synthesized at Genepharma Company. After 48 h, transfected cells were harvested. The sequences of all above plasmids were provided in Table 2.

**Table 2:** The sequences of plasmids applied for cell transfection

Plasmids	Sequences (5′–3′)
sh-NC (for SNHG10)	CCGGACATGACTGTATCTGTCTAGTCTCGAGACTAGACAGATACAGTCATGTTTTTTG
sh-SNHG10#1	CCGGGCAACCGCTTTGTTAGTTAATCTCGAGATTAACTAACAAAGCGGTTGCTTTTTG
sh-SNHG10#2	CCGGGCGCGCGATTATTTCTCTAGACTCGAGTCTAGAGAAATAATCGCGCGCTTTTTG
sh-NC (for FBXL19)	CCGGCGAGACATAGGATCGGCAGATCTCGAGATCTGCCGATCCTATGTCTCGTTTTTG
sh-FBXL19#1	CCGGGCCGAGACATGAAGAAGTTCGCTCGAGCGAACTTCTTCATGTCTCGGCTTTTTG
sh-FBXL19#2	CCGGGCTGGATCGAGGATGTTAAAGCTCGAGCTTTAACATCCTCGATCCAGCTTTTTG
sh-NC (for ETS1)	CCGGATCAGAGTACTCCTACGCTGACTCGAGTCAGCGTAGGAGTACTCTGATTTTTTG
sh-ETS1#1	CCGGGCAACTCAGGAAGTTCCTACTCTCGAGAGTAGGAACTTCCTGAGTTGCTTTTTG
sh-ETS1#2	CCGGGGAAGTTCCTACTGGTCTTGACTCGAGTCAAGACCAGTAGGAACTTCCTTTTTG
NC mimics/inhibitor	CCAAGCCACCCUACCGCUUGCA
miR-532-3p mimics	CCUCCCACACCCAAGGCUUGCA
miR-532-3p inhibitor	UGCAAGCCUUGGGUGUGGGAGG
pcDNA3.1/SNHG10	AGCCTCATCCTACTGCCTTACTATTGGTCGTCGGCAACCGCTTTGTTAGTTAATTGGAGAGACTGGATGTCCATCAGGGTCACGCTTGCAGGGCAACGAGAGGCAAAACAAGAGGGAAGACGACTTTCCTCCTGTGAGCCAATGAGGCCAGCTGGACTACGCCGAGACAACTGGGAGAGGCGCGGGACTCGCCCGTTCCGCGGAACGCCGGGAAGGGGTCACCTCCTGATGAAGTTTCCGGTTCCGGTGTCAGCGGCGGTTGAATTGCCATGGCAATGCGGTGGGCGCGCGCTTGTCGTGTTGGTCTCTTGGGAGGTAGCGGGGCTAGGCCGGGCGGGTATCCGCCTCTCCCAGCTTAGGTGAGCGTCCCCGGGCGCCTCCGGAGCGCCGCGGCCGCATGCAGTTCGTCGTGGCGGGGAGCCGGAGCCTGACCGGGGTTCCAGCGCTCGGGCCGTAGCCTTGGCTCCTGGACTTTCCCTGGCTCCGCCGCCACGTGGGAGCTGAGGCTCTGGGGCTTCCGCCTCCGGCGCGCGATTATTTCTCTAGAACAGTTTTCATTTTTAAAATTTGTAAAGCGCTTTTGCCTGTGTGATTTCCTCTGGGTTTTTTTTTTTTTTCTTCCTTTTTGTAGAGACGGAATTGGCGGCGGGGGCGGGGGGTCGATGTCTCACTTTTTTGCCCAGGCTGGTCTCGAACTCCTGGCTTCAAGGGATCCTCCTGCCTCGGCCTCTTAAAGTGCTGGGATTACAGGCGTGAGCCACCGCCCCCGGCCGCCTCTGAGTTTCCAGCCTCGTTGGCCCTCCAGCCTTTTAACCTGTTGGGCCTAGGATCAGGAAAGGTTTGTTGAATGGGGAACTAAGAAGTGAATTCGTTCGTTCGACAAACGTTTCCTGAGCAGCCGCTGGGTGCTAGGCGCAGTGCCAGCGCGGAATGTCCAGGGAGACCTGGTGCCCAAAGCTTGGACCCATCGTGAGAAATGAGAAGCAGATACAAAGCAGTGTGGGAGTGCAGAGGAGACAAAGCAAGCCTCATCAGGCCCATTGCTTGCTCTGCTCTCCCTTGTACTTACCAGTGCTTGACAATATACAGTTATTTACTAGCTTGGTTATTGACTTCCTACCCAGCACTCAGTTTTATTCACTGCTGTATCCTCAGTGCCTAGGACGATGCTTGGAACGTGGTAAGTGCTCCTATTGGCGGGAAGAATAAATCCGGAAGAGCAGGACCAGTGGACTTGCTACATAATCTGTAGTCTTGGAGCCGCACAGGGTTGGTGGTACCCTCGAGCACACCAGACTTGCAGAAAAAGCATACTCCAGAGGAAGCTGAGGCATGCCTGCTCGAGAGCCAGCTGTTCCATGTGCAATTTTCCTCTGATAGTTTCTGGTCACTGTTGCCACGGTGATAATGACTGGGCTATGTCATTATCTATCCGCCAACAGTAAGAGAAGCTTTGCAGTCGAGATATTGTTTAGCAGATGGAGTGTTTTCTGTTGAACACTAAGTACTGCCACAAGTTACTTTTTTTTTTTTAAACTTTGAGTATTTTTTTACAATGTTGCTGGAGGTGATCTGTTTATGCTTTGAGAGTGTTCGAATTTAAAATCAGAAAATCATGTCAGTGAGTGAGTCTTTCAAATAATCCTTCGGCATGAAACCTGAGCCTAGTAAACTATGAAAGTAAACTCGGCACATTACCCGAAAGTCTCAATGTCATATTTTCACCCCCATCAATATTATTGATGATTGCTCATTTTCTAATGTGGGACCTGAAATTTACCAGGTGCTTAAAGAATCTTTTTGTTTTTCAGATTCATTGATTCCAGGTAAATCAGAGGAACAAGCAACATGAACAGAAATATGTAGAAAAAGCTATTATGCAGAAGCATAATTGTTGTTTCAGAAGTCCAGCATCTGGTGCACTTAACAATAGAGAATATATTAAACTCTTTCCAAAATAAAAAAAAAAAAAAAAAAAAA
pcDNA3.1/FBXL19	ATGGGTATGAAAGTCCCCGGAAAGGGGGAATCTGGGCCCTCGGCGTTGCTGACGCCCCCAATGTCGTCGAGCAGCCGGGGGCCGGGGGCCGGAGCGCGCCGACGCCGAACCCGCTGCCGCCGCTGCCGGGCCTGTGTGCGAACTGAGTGCGGGGATTGCCACTTCTGCCGAGACATGAAGAAGTTCGGGGGGCCCGGGCGCATGAAGCAGTCGTGCCTGCTCCGGCAGTGCACTGCCCCCGTGCTCCCACACACAGCTGTGTGCCTCTTGTGTGGGGAGGCTGGGAAGGAGGACACGGTGGAGGGAGAGGAAGAGAAATTTGGTTTGAGCCTCATGGAGTGTACAATCTGCAACGAGATCGTCCACCCCGGCTGCCTGAAGATGGGGAAGGCTGAGGGTGTCATCAATGCAGAGATCCCCAACTGCTGGGAGTGCCCTCGCTGCACCCAGGAAGGCCGCACCAGCAAGGATTCAGGTGAGGGGCCTGGCCGCCGTAGGGCCGACAACGGCGAGGAGGGCGCCAGCTTGGGGAGCGGATGGAAGCTGACAGAGGAGCCACCGCTTCCACCGCCCCCGCCCAGGCGCAAGGGCCCCCTGCCTGCCGGGCCCCCCCCGGAGGACGTGCCTGGGCCCCCCAAACGAAAGGAAAGGGAGGCAGGGAATGAGCCTCCCACCCCAAGGAAAAAGGTGAAAGGAGGCCGAGAGAGGCACCTGAAGAAGGTGGGTGGAGACGCCTGCCTCCTCCGAGGATCGGACCCAGGCGGCCCGGGCCTGCTGCCCCCCAGGGTTCTGAATCCGAGCCAGGCTTTCTCATCCTGCCACCCTGGGCTCCCTCCCGAGAACTGGGAGAAACCAAAGCCGCCTTTGGCCTCTGCAGAGGGCCCAGCGGTGCCGTCCCCGTCCCCGCAGAGGGAGAAGCTAGAGCGTTTCAAGCGGATGTGCCAGCTGCTGGAACGGGTGCCTGACACCTCCTCTTCCTCCTCGGACTCAGACTCCGACTCCGACTCTTCGGGCACATCGCTGAGTGAGGACGAAGCCCCCGGCGAGGCCCGGAATGGGCGACGGCCAGCCCGGGGCAGCTCTGGCGAGAAGGAGAACCGTGGGGGGCGGCGGGCTGTGCGCCCTGGCAGTGGGGGGCCCCTACTCAGCTGGCCCCTGGGCCCAGCCCCACCACCCCGGCCTCCACAGCTGGAGCGGCACGTGGTGCGGCCCCCGCCTCGAAGCCCTGAGCCCGACACACTCCCCTTGGCTGCTGGATCCGACCACCCCCTGCCCCGGGCCGCCTGGCTTCGCGTCTTCCAGCACCTCGGGCCGCGGGAGCTGTGTATCTGCATGCGAGTCTGCCGAACTTGGAGCCGCTGGTGCTATGACAAGCGTCTGTGGCCTCGAATGGACCTGAGCCGGCGGAAGTCACTGACCCCGCCCATGCTCAGTGGTGTGGTTCGCCGCCAGCCCCGTGCCCTGGACCTCAGCTGGACAGGTGTCTCCAAGAAGCAGCTCATGTGGCTTCTGAACCGACTACAAGGCCTGCAGGAGCTGGTGCTCTCTGGCTGCTCCTGGCTCTCTGTCTCTGCCCTGGGCTCAGCCCCACTGCCAGCCCTGCGGCTCCTGGACCTCCGCTGGATCGAGGATGTTAAAGACTCCCAGCTCCGGGAGTTGCTGCTGCCTCCACCAGACACCAAACCAGGGCAAACAGAGAGCCGTGGTCGGCTGCAGGGGGTGGCAGAACTGCGTCTGGCAGGTTTGGAGCTGACAGATGCCTCCCTGCGTCTCCTGCTGCGTCACGCACCCCAGCTGAGCGCCCTGGACCTGAGCCACTGCGCCCACGTCGGGGACCCCAGTGTTCACCTCCTCACGGCCCCCACGTCCCCACTCCGCGAGACCCTGGTGCACCTCAATCTTGCTGGTTGCCACCGCCTAACGGACCACTGCCTCCCGCTGTTCCGCCGCTGCCCTCGTCTACGCCGCCTAGACCTGCGCTCCTGCCGCCAGCTCTCACCCGAAGCTTGTGCCCGGCTGGCAGCTGCCGGGCCCCCTGGCCCCTTCCGCTGCCCTGAGGAGAAGCTGCTTCTCAAGGACAGCTAG

### Colony formation assay

Clonogenic U138 and U251 cells were prepared in 6-well plate for 14-day, with 500 cells/well. The colonies were visualized via staining in 0.5% crystal violet solution after fixation, followed by counting manually.

### EdU staining

U138 and U251 cells were added to 96-well plate containing EdU medium diluent and cultured for 3 h in line with the instruction (Ribobio, Guangzhou, China). Following being fixed and permeabilized, cell nuclei were counterstained with DAPI and then the stained cells were observed using fluorescent microscope (Nikon, Tokyo, Japan).

### Caspase activity detection

Using the caspase-3/8/9 activity kits, the detection of caspase-3/8/9 activities were achieved as instructed by supplier (Abcam, Cambridge, MA). After incubation for 2 h, absorbance at 405 nm was detected by utilization of the ELx 800 microplate reader (Bio-Tek, Vermont, USA).

### TUNEL staining

TUNEL staining in U138 and U251 cells were implemented with the direction provided by manufacturer (Beyotime, Shanghai, China). After DAPI staining, the TUNEL-positive cells were determined using fluorescent microscope (Nikon, Tokyo, Japan).

### Sphere formation assay

U138 and U251 cells were seeded to each well of the 96-well ultralow attachment plates (Corning Inc., New York, NY), and then the sphere medium was added for 7 days of incubation. After that, the spheres were imaged and counted under a light microscope (Olympus, Tokyo, Japan).

### Wound healing assay

Cell samples were seeded to 6-well plates and cultivated until 90% confluence, and then the artificial wounds were created by 200 μL of pipette tip. After that, cells were cultured with serum-free medium, and then the images of wound closure at 0 and 24 h were acquired using microscope (Olympus, Tokyo, Japan).

### Transwell invasion assay

Transwell chamber was pre-coated with Matrigel (BD Biosciences, Franklin Lakes, NJ) for cell invasion assay. The transfected cell samples (1 × 10^5^) were added to the upper chamber with serum-free medium, and the lower chamber was filled with 500 μL of complete culture medium. After 24 h of incubation, the chamber was fixed by 4% PFA and stained by crystal violet solution. Invading cells were finally counted in 5 random fields under a microscope (Olympus, Tokyo, Japan).

### Western blot

Cells were treated with RIPA lysis buffer for the isolation of total proteins. Following, the proteins were separated on 12% SDS-PAGE and then shifted to PVDF membranes. After blocking by 5% nonfat milk, membranes were probed all night at 4 ℃ with the primary antibodies against FBXL19 (ab172961), NPTN (ab272652), SRRM2 (ab182251), OCT4 (ab19857), Nanog (ab109250), SOX2 (ab97959) and the loading controls GAPDH (ab9485) and Cofilin (ab124979). Following washing in TBST, membranes were probed with the HRP-labelled secondary antibodies (ab205718) for 2 h at room temperature. All of the antibodies were procured from Abcam (Cambridge, MA). The protein signal was studied using the enhanced chemiluminescence system (ECL; Santa Cruz Biotechnology, Santa Cruz, CA).

### In vivo tumor formation assay

BALB/c nude mice (6 weeks old) were procured from Slac Laboratories (Shanghai, China) and used for in vivo assay. The animal-related study was undertaken with the approval of the Animal Research Ethics Committee of the First People’s Hospital of Yunnan Province. In vivo tumor formation assay was implemented via subcutaneous injection of 5 × 10^6^ cells transfected with sh-SNHG10#1 or sh-NC into nude mice. Tumor volumes were monitored every 4 days. 28-days later, mice were sacrificed and tumors were carefully excised and weighted for further analysis.

### Immunohistochemistry (IHC)

The tumor tissues collected from in vivo tumor formation assay were fixed using 4% PFA, dehydrated and then embedded in paraffin. Afterwards, the consecutive sections at 4 μm thick were prepared for IHC assay by use of antibodies (Abcam) against Ki-67 (ab16667) and PCNA (ab29).

### Subcellular fractionation

Total 1 × 10^6^ U138 and U251 cells were centrifuged to collect the supernatant after washing in PBS. The nuclear or cytoplasmic RNAs were separately isolated using PARIS™ Kit (Invitrogen). RNA expression level was tested by RT-qPCR.

### Fish

After being fixated, U138 and U251 cells were prepared for culturing in hybridization buffer with the SNHG10-FISH probe (Ribobio) and then dehydrated. Hoechst solution was applied for nuclear detection. Cells were finally assayed via fluorescent microscope (Nikon, Tokyo, Japan).

### RNA immunoprecipitation (RIP)

The cell lysates were acquired via RIP lysis buffer after washing in precooled PBS. The magnetic beads-bound antibodies against human Ago2 (ab186733, Abcam) and control normal IgG (ab19047, Abcam) were prepared for incubation with the cell lysates in RIP buffer, followed by RNA analysis via RT-qPCR.

### RNA pull down assay

The lysates from U138 and U251 cells were extracted by RIPA lysis buffer and collected for cultivation with the biotinylated RNA probes. The miR-532-3p fragments with wild-type or mutated seed region sequences were synthesized and biotinylated into Bio-miR-532-3p-WT/Mut, with a biotin-labeled nonsense RNA sequence as the negative control. Relative RNA enrichment was monitored by RT-qPCR.

### Luciferase reporter assay

The full-length SNHG10 or FBXL19 3′UTR fragments with wild-type or mutated miR-532-3p target sequences were inserted into the luciferase reporter vector pmirGLO (Promega, Madison, WI), termed SNHG10-WT/Mut and FBXL19-WT/Mut. The co-transfection of SNHG10-WT/Mut or FBXL19-WT/Mut with miR-532-3p mimics or NC mimics into U138 and U251 was performed via Lipofectamine 2000. Besides, wild-type or mutant type of SNHG10 promoter was sub-cloned into the pGL3 luciferase reporter vector (Invitrogen) for establishing promoter WT/Mut vector, which was then separately co-transfected with pcDNA3.1 or pcDNA3.1/ETS1. The relative luciferase was determined by analysis of Luciferase Reporter Assay System (Promega) after 48 h of transfection.

### Chromatin immunoprecipitation (ChIP)

In line with the manufacturer’s protocol, ChIP assay conducted in U138 and U251 cells was achieved by the application of EZ-ChIP Kit (Millipore, Massachusetts, USA). The cross-linked chromatin was sonicated and then mixed with the anti-ETS1 antibody (#14,069, Cell signaling technology, Boston, USA) or control IgG antibody (#3900, Cell signaling technology) for immunoprecipitation. ChIP-derived DNA fragments were assayed via RT-qPCR after being recovered by magnetic beads.

### Statistical analyses

All results were exhibited with the method of mean ± standard deviation (S.D.) and analyzed with the application of Prism 5.0 software (GraphPad Software, Inc., La Jolla, CA). The measurement data of independent bio-triplicates were processed by Student’s t-test or ANOVA, with P < 0.05 as the significant level.

## Results

### SNHG10 was up-regulated and exerted oncogenic functions in glioma

**Fig. 1:**
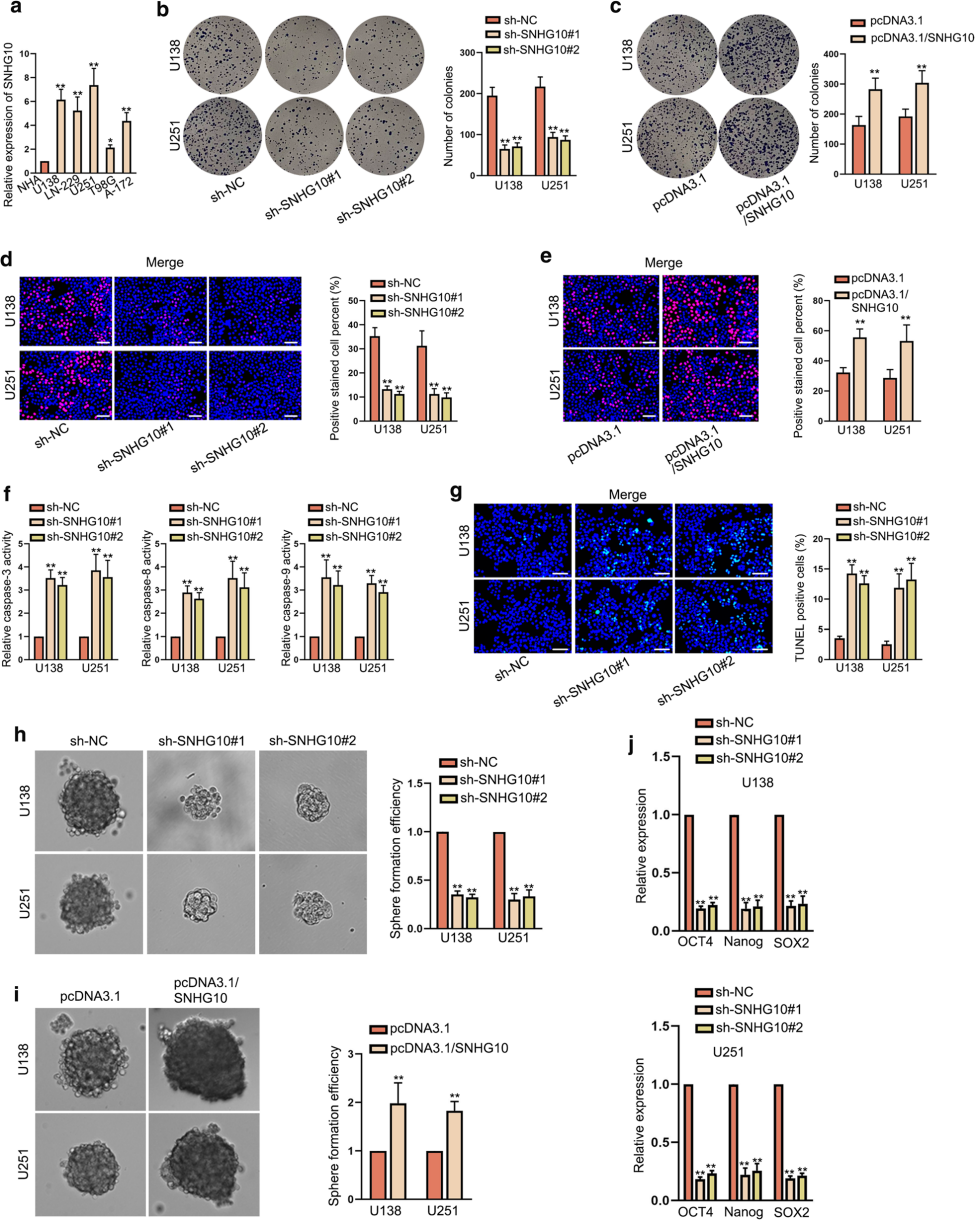
SNHG10 was up-regulated and exerted oncogenic functions in glioma. **a** SNHG10 expression was detected in glioma cell lines (U138, LN-229, U251, T98G and A-172) and normal NHA cells. **b**, c Colony formation assay examined the effect of SNHG10 inhibition or overexpression on glioma cell proliferation. **d, e** EdU assays assessed the proliferation of glioma cells with SNHG10 inhibition or overexpression. Scale bar = 100 μm. **f** Caspase-3/8/9 activities were examined under SNHG10 deficiency. **g** TUNEL assay appraised the apoptosis of cells transfected with sh-SNHG10#1/2. Scale bar = 100 μm. **h–j** Sphere formation efficiency and the expressions of three stemness-related markers (OCT4, Nanog, and SOX2) in indicated groups were evaluated by sphere formation assays and RT-qPCR. *P < 0.05, **P < 0.01

To get the basic information of SNHG10 in glioma, the first step was to detect its expression. RT-qPCR data revealed that SNHG10 expression was conspicuously high in glioma cell lines (U138, LN-229, U251, T98G and A-172) compared with normal NHA cells (Fig. 1a). On this basis, U251 and U138 cells were selected to conduct following experiments for that they presented higher SNHG10 expression than other cancerous cells. To know the impact of SNHG10 on glioma cell functions, SNHG10 expression was silenced or up-regulated in U138 and U251 cells through separately transfecting sh-SNHG10#1/2 or pcDNA3.1/SNHG10 (Additional file 1: Fig. S1A, B). Subsequently, we discovered that the down-regulation of SNHG10 decreased the colony formation ability while up-regulation of SNHG10 increased such ability in both the two kinds of glioma cells (Fig. 1b, c). Additionally, the results of EdU assays further verified that cell proliferation was curbed by down-regulated SNHG10 and facilitated by overexpressed SNHG10 (Fig. 1d, e). Regarding to cell apoptosis, it was unveiled that the activities of caspase-3, caspase-8 and caspase-9 were enhanced by SNHG10 silencing (Fig. 1f). Moreover, TUNEL assay results displayed that the apoptosis rate of glioma cells was remarkably elevated in response to the down-regulation of SNHG10 (Fig. 1g). Besides, we observed that SNHG10 knockdown impaired the efficiency of sphere formation while up-regulation of SHNG10 exerted the opposite effect (Fig. 1h, i). Later, the expression of stem cell markers was examined by RT-qPCR. Results uncovered that expression levels of OCT4, Nanog and SOX2 were dropped upon SNHG10 down-regulation and fortified under SNHG10 overexpression (Fig. 1j, and Additional file 1: Fig. S1C). Furthermore, we also analyzed whether SNHG10 had influences on cell migration and invasion. The outcomes of wound healing assay unveiled that the migratory ability was refrained in cells transfected with sh-SNHG10#1/2 whereas hastened in those with pcDNA3.1/SNHG10 transfection (Additional file 1: Fig. S1D, E). Likewise, SNHG10 knockdown hampered cell invasion while its overexpression accelerated cell invasion in glioma (Additional file 1: Fig. S1F, G). Taken all together, SNHG10 contributed to the malignant behaviors of glioma cells in vitro.

### Loss of SNHG10 blocked tumor growth of glioma in vivo

**Fig. 2:**
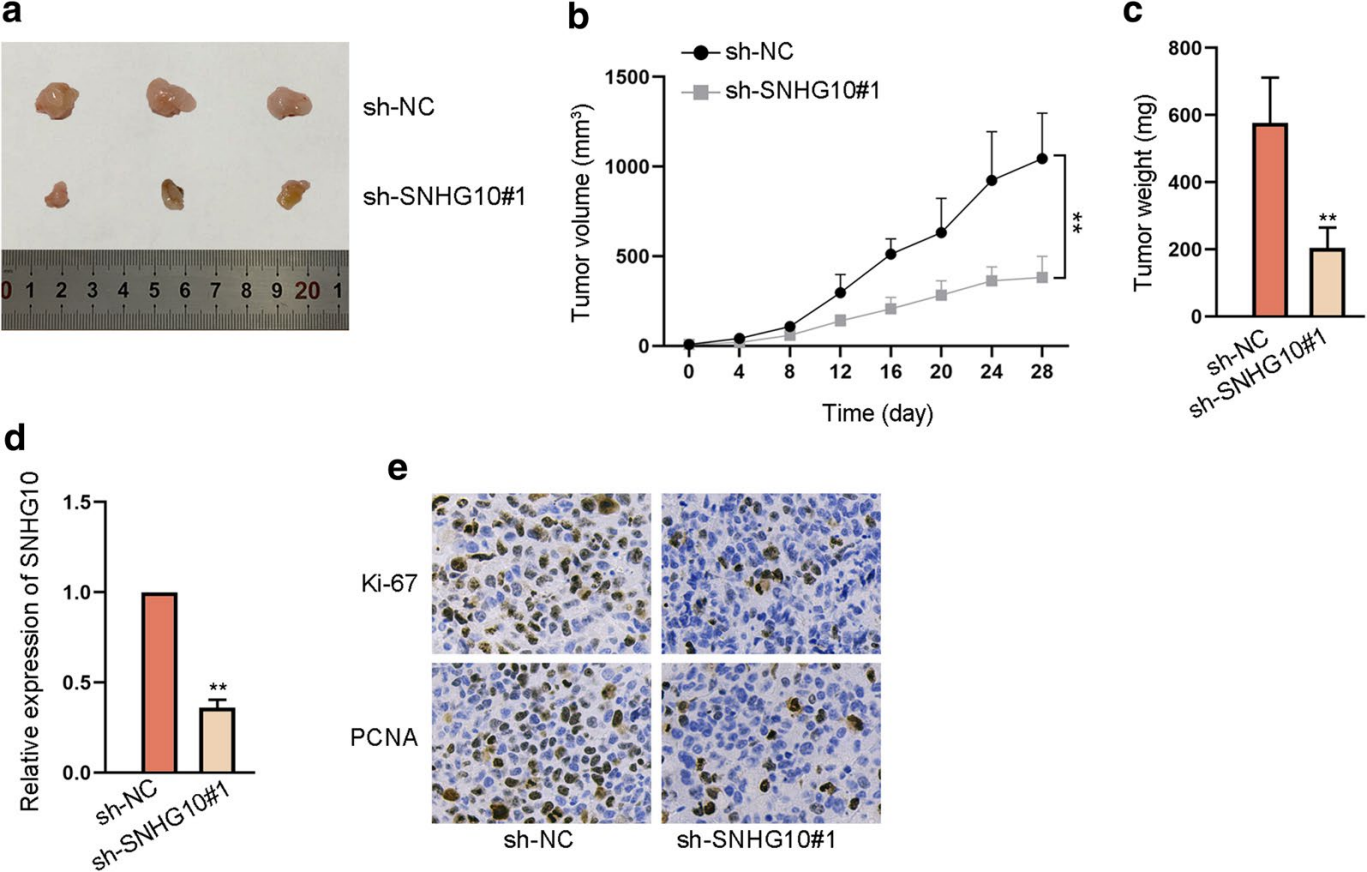
Loss of SNHG10 restrained tumor growth in vivo. **a** The tumors excised from mice with subcutaneous injection of sh-SNHG10#1 and sh-NC transfected cells. **b**, **c** The growth curve and the weight of excised tumors in sh-SNHG10#1 and sh-NC groups were determined. **d** RT-qPCR tested SNHG10 expression in the tumors from mice in sh-NC and sh-SNHG10#1 groups. **e** IHC assay evaluated Ki-67 and PCNA expression in tumors with or without SNHG10 inhibition. **P < 0.01

To explore the influence of SNHG10 on gliomagenesis, we also performed in vivo assays using nude mice. As shown in Fig. 2a, the tumors in sh-SNHG10#1 group were smaller than those in sh-NC group. We also observed that the growth of tumors was obstructed and the weight of them was reduced under SNHG10 depletion (Fig. 2b, c). Then, RT-qPCR manifested that SNHG10 expression in tumors from glioma cells with sh-SNHG10#1 transfection was lowered compared to that in those from control group (Fig. 2d). In addition, the positive staining of Ki-67 and PCNA was also reduced in tumors with silenced SNHG10 (Fig. 2e). In a word, SNHG10 promoted tumor growth of glioma in vivo.

### MiR-532-3p sponged by SNHG10 hampered the growth and stemness of glioma cells

**Fig. 3:**
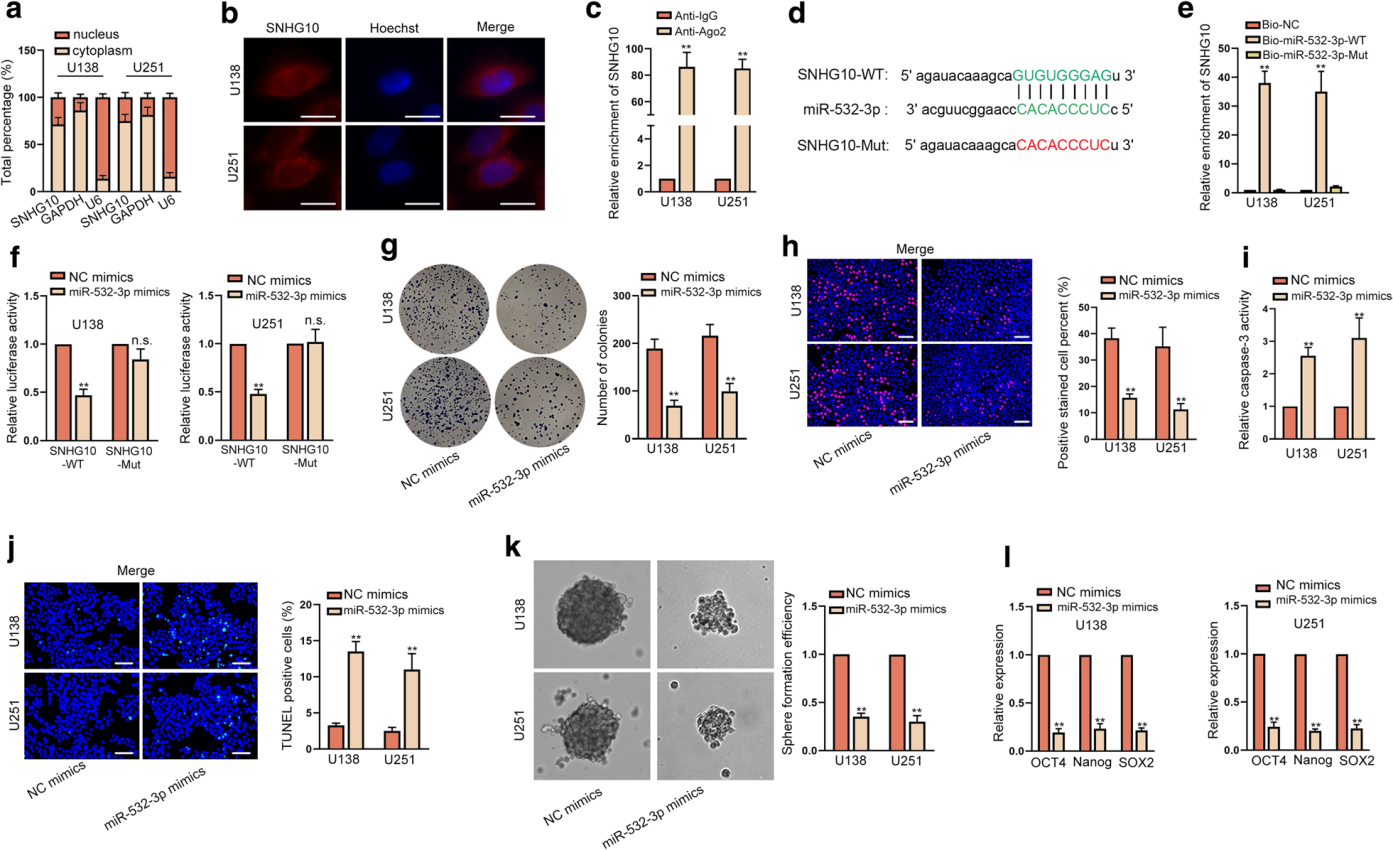
MiR-532-3p was sponged by SNHG10 and hampered cell growth and stemness in glioma. **a, b** Subcellular fractionation and FISH assays (scale bar = 10 μm) were performed to judge the subcellular localization of SNHG10 in U138 and U251 cells. **c** RIP assays demonstrated that SNHG10 could be enriched in the beads conjugated with Ago2 antibody. **d** Binding sites between SNHG10 and miR-532-3p were shown. **e** RNA pull down assay validated that SNHG10 could bind to miR-532-3p. **f** Luciferase reporter assay certified the impact of miR-532-3p on the luciferase activity of SNHG10-WT/Mut. **g**, **h** The proliferation of cells with or without miR-532-3p elevation was examined by colony formation and EdU assays (scale bar = 100 μm). **i**, **j** Cell apoptosis was probed by caspase-3 activity detection and TUNEL assay (scale bar = 100 μm) upon miR-532-3p overexpression. **k**, **l** Sphere formation assay and RT-qPCR examined the effect of overexpressed miR-532-3p on stemness. **P < 0.01, n.s. presented no significance

Next, we wanted to know the mechanism underlying SNHG10-mediated influences on glioma cell functions. Hence, subcellular fractionation and FISH assays were exploited to assess the localization of SNHG10 in glioma cells. Results depicted that SNHG10 was mainly amassed in cytoplasm rather than in nucleus (Fig. 3a, b). Besides, RIP assay results disclosed that SNHG10 exhibited strong enrichment in Ago2 group relative to IgG group (Fig. 3c), implying that SNHG10 might function as a ceRNA in glioma. Subsequently, starBase (https://starbase.sysu.edu.cn) predicted miR-532-3p and miR-2278 as the potential miRNAs for SNHG10. We then evaluated the expression of these two miRNAs in glioma cells. As a result, miR-532-3p presented low expression while miR-2278 showed no conspicuous changes in glioma cells compared with normal controls (Additional file 1: Fig. S1H). Thus, miR-532-3p was chosen for the subsequent study. The potential binding sites between SNHG10 and miR-532-3p were displayed in Fig. 3d. RNA pull down revealed that biotinylated miR-532-3p-WT could pull down SNHG10 but biotinylated miR-532-3p-Mut failed (Fig. 3e). By transfecting miR-532-3p mimics, we obtained augmented expression of miR-532-3p in U138 and U251 cells (Additional file 1: Fig. S1I). Results of luciferase reporter assay manifested that the luciferase activity of SNHG10-WT that could recognized by miR-532-3p was decreased by miR-532-3p up-regulation, whereas that of SNHG10-Mut which could not interact with miR-532-3p was not affected by elevated miR-532-3p (Fig. 3f). Thereafter, we investigated the functional role of miR-532-3p in glioma. Results from colony formation and EdU assays confirmed that cell proliferation was dramatically suppressed by upregulated miR-532-3p (Fig. 3g, h). Otherwise, the apoptosis of glioma cells was boosted due to miR-532-3p up-regulation (Fig. 3i, j and Additional file 1: Fig. S1J). Additionally, ectopic expression of miR-532-3p caused a decline in sphere formation efficiency and in the levels of stemness-related markers (Fig. 3k, l). In short, miR-532-3p could bind to SNHG10 and hinder the progression of glioma.

### FBXL19 was negatively regulated by miR-532-3p

**Fig. 4:**
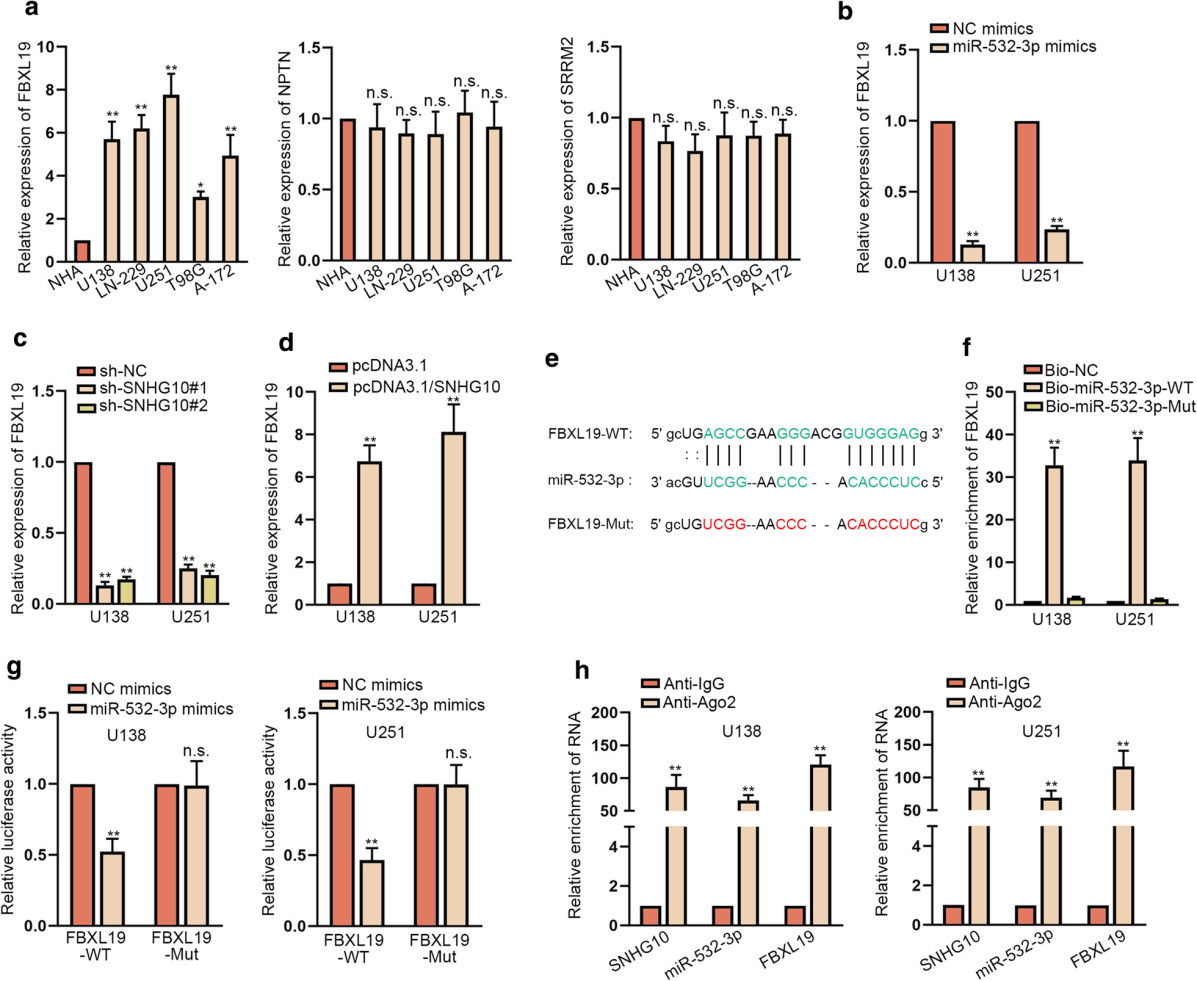
FBXL19 was negatively regulated by miR-532-3p. **a** The expression levels of three predicted mRNAs targeted by miR-532-3p were evaluated via RT-qPCR. **b** FBXL19 expression was measured by RT-qPCR in cells transfected with NC mimics or miR-532-3p mimics. **c**, **d** FBXL19 expression was evaluated by RT-qPCR in cells transfected with sh-SNHG10#1/2 or pcDNA3.1/SNHG10. **e** Binding sites between FBXL19 and miR-532-3p were shown. **f** RNA pull down experiments validated that FBXL19 could bind to miR-532-3p. **g** Luciferase reporter assay certified the influence of miR-532-3p on the luciferase activity of FBXL19-WT/Mut. **h** RIP validated that SNHG10, miR-532-3p and FBXL19 were co-existed in Ago2-assembled RNA induced silencing complexes (RISCs). *P < 0.05, **P < 0.01, n.s. presented no significance

It is well known that miRNAs could modulate the expression of target genes via binding to their 3′UTR. As predicted by starBase, FBXL19, NPTN and SRRM2 were putative mRNAs that could be recognized by miR-532-3p. RT-qPCR results exhibited that only FBXL19 among the three targets was evidently up-regulated in glioma cells compared to normal controls (Fig. 4a), which further confirmed by western blot (Additional file 2: Fig. S2A). Later, we noticed a sharp decline in FBXL19 mRNA expression and protein level after transfecting miR-532-3p mimics or sh-SNHG10#1/2 into U138 and U251 cells (Fig. 4b, c and Additional file 2: Fig. S2B, C). On the contrary, FBXL19 mRNA and protein expressions were both elevated in glioma cells facing the transfection with pcDNA3.1/SNHG10 (Fig. 4d and Additional file 2: Fig. S2D). Furthermore, the binding sequence between FBXL19 and miR-532-3p was shown in Fig. 4e. RNA pull down experiments demonstrated the binding of miR-532-3p to FBXL19 in both cells (Fig. 4f). Luciferase reporter assay results certified that elevating the expression of miR-532-3p could lessen the luciferase activity of FBXL19-WT since it could bind to miR-532-3p, while had no impact on that of FBXL19-Mut as it lost the ability to interact with miR-532-3p (Fig. 4g). Moreover, RIP assays displayed that SNHG10, miR-532-3p and FBXL19 were all remarkably enriched in the beads conjugated with Ago2 antibody but not in those with IgG antibody (Fig. 4h). In brief, FBXL19 was the downstream target of miR-532-3p in glioma.

### FBXL19 functioned as an oncogene in glioma

**Fig. 5:**
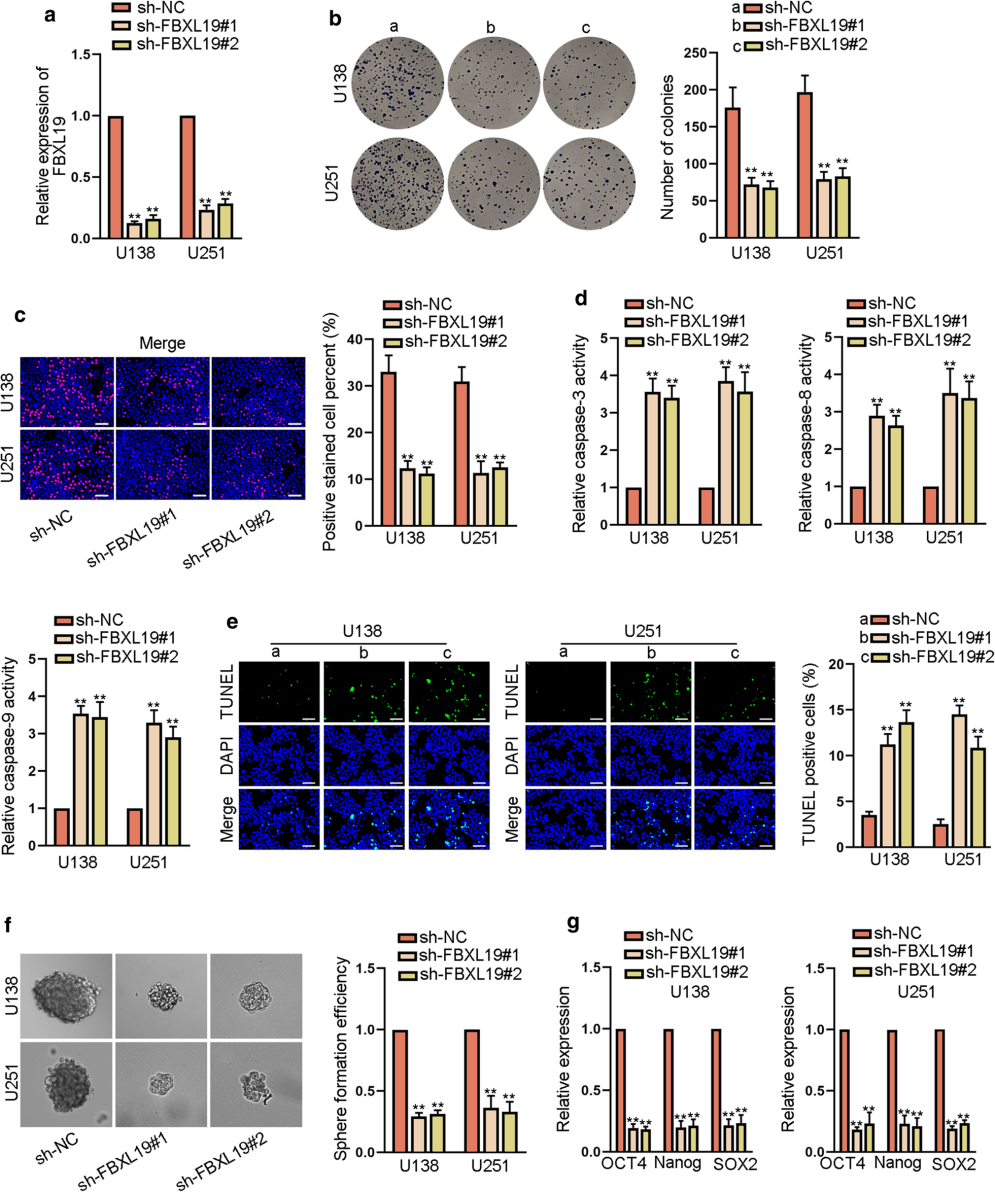
FBXL19 acted as an oncogene in glioma. **a** FBXL19 expression was examined by RT-qPCR in cells transfected with sh-FBXL19#1/2. **b, c** The proliferation was evaluated via colony formation and EdU assays (scale bar = 100 μm) in cells with the transfection of sh-FBXL19#1/2. **d, e** Cell apoptosis was assessed in different groups by caspase 3/8/9 activity detection and TUNEL assay (scale bar = 100 μm). **f, g** The stemness was appraised by sphere formation assay and RT-qPCR analysis of stemness-associated marker expressions in cells with orwithout -FBXL19 inhibition. **P < 0.01

Then, we analyzed the effect of FBXL19 on the development of glioma. At the beginning, we used sh-FBXL19#1/2 to knock down the expression of FBXL19 in U138 and U251 cells (Fig. 5a). The results of colony formation and EdU assays revealed that cell proliferation was slumped by FBXL19 silencing (Fig. 5b, c). In addition, caspase-3, caspase-8 and caspase-9 activities were all enhanced by down-regulation of FBXL19 (Fig. 5d). As detected by TUNEL assays, the apoptosis of glioma cells was promoted due to the absence of FBXL19 (Fig. 5e). Meanwhile, sphere formation efficiency as well as the expression of OTC4, Nanog and SOX2 was apparently reduced by down-regulated FBXL19 (Fig. 5f, g and Additional file 2: Fig. S2E). In conclusion, FBXL19 knockdown could inhibit the development of glioma.

### SNHG10 facilitated the progression of glioma via up-regulating FBXL19 expression

**Fig. 6:**
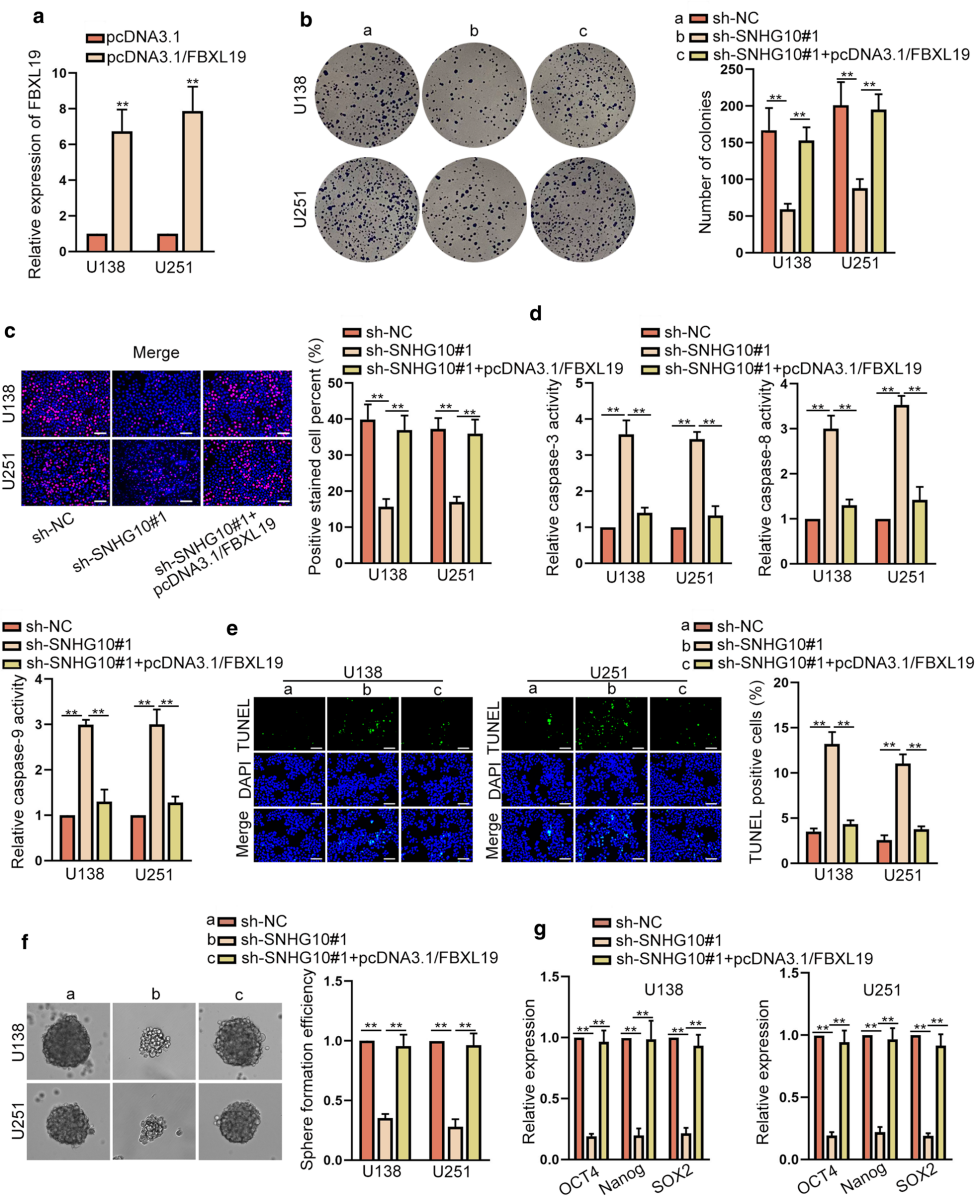
SNHG10 facilitated the progression of glioma via up-regulating FBXL19 expression. **a** FBXL19 expression was examined by RT-qPCR in cells with the transfection of pcDNA3.1/FBXL19. **b**, **c** The proliferation of cells transfected with sh-NC, sh-SNHG10#1 or sh-SNHG10#1 + pcDNA3.1/FBXL19 was evaluated through colony formation and EdU assays (scale bar = 100 μm). **d**, **e** Cell apoptosis was examined via caspase activity detection and TUNEL assay (scale bar = 100 μm) in different groups. **f**, **g** The stemness in each group was measured by sphere formation assay and RT-qPCR analyses. **P < 0.01

Furthermore, we designed and conducted rescue assays to validate the regulatory role of SNHG10/miR-532-3p/FBXL19 axis in glioma. After transfecting with pcDNA3.1/FBXL19, we observed the augmented FBXL19 expression in U138 and U251 cells (Fig. 6a). As expected, the falling trend of cell proliferation imposed by SNHG10 down-regulation was reversed in response to the up-regulation of FBXL19 (Fig. 6b, c). Also, the ascending caspase-3/8/9 activities induced by down-regulated SNHG10 was restored by FBXL19 overexpression (Fig. 6d). The cell apoptosis detected via TUNEL assays also presented similar trends under the same conditions (Fig. 6e). Moreover, the inhibited sphere formation abilities and controlled expressions of stemness-associated markers caused by SNHG10 silencing were both retrieved by up-regulation of FBXL19 (Fig. 6f, g). In the meantime, SNHG10 knockdown-induced suppressive effects on cell migration and invasion were also countervailed by FBXL19 overexpression (Additional file 2: Fig. S2F, G). In a word, SNHG10 depended on FBXL19 to accelerate the malignant behaviors of glioma cells.

### ETS1 promoted the transcription of SNHG10 in glioma

**Fig. 7:**
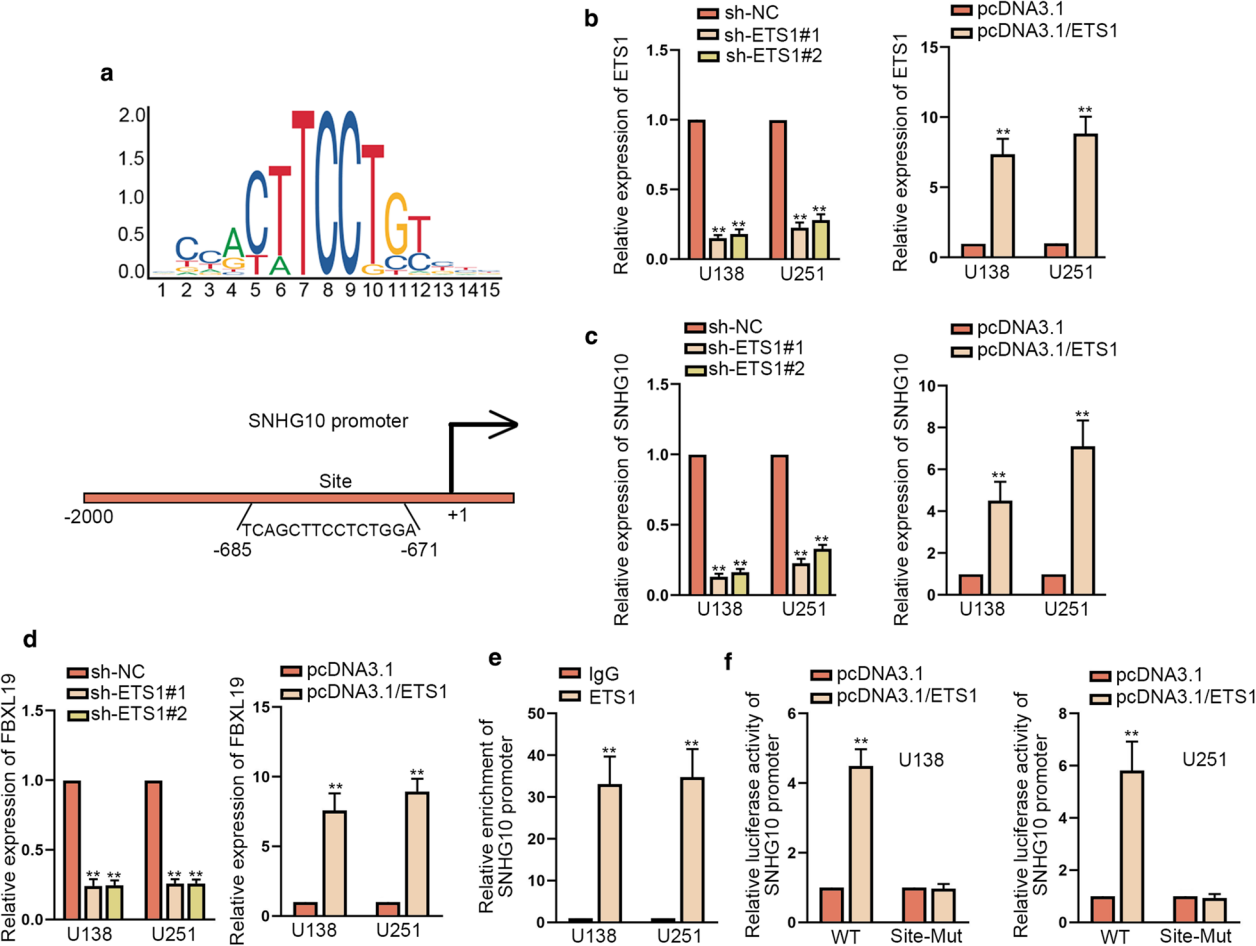
ETS1 promoted the transcription of SNHG10 in glioma. **a** JASPAR predicted the DNA binding motif of ETS1 and the possible binding sites of ETS1 in SNHG10 promoter. **b** ETS1 expression was tested by RT-qPCR in cells transfected with sh-ETS1#1/2 or pcDNA3.1/ETS1. **c**, **d** SNHG10 or FBXL19 expression was evaluated via RT-qPCR in cells with ETS1 suppression or overexpression. **e** ChIP assays validated the interaction between SNHG10 promoter and ETS1. **f** Luciferase reporter assays verified that ETS1 bound to SNHG10 promoter at the putative sites. **P < 0.01

Finally, we explored the upstream of SNHG10 in glioma cells. By utilizing UCSC (https://genome.ucsc.edu/) and JASPAR (https://jaspar.genereg.net/), we found the motif of ETS1 and the underlying binding sites of ETS1 in SNHG10 promoter (Fig. 7a). For further study, the successful knockdown or overexpression of ETS1 respectively by sh-ETS1#1/2 or by pcDNA3.1/ETS1 were confirmed by RT-qPCR (Fig. 7b). Subsequently, RT-qPCR revealed that SNHG10 expression, as well as FBXL19 expression, was reduced due to ETS1 knockdown and up-regulated in response to ETS1 overexpression (Fig. 7c, d). The outcomes of ChIP assay showed that SNHG10 promoter was significantly enriched by ETS1 antibody rather than IgG antibody (Fig. 7e). Luciferase reporter assay results exhibited that ETS1 up-regulation elevated the luciferase activity of SNHG10-WT promoter but not that of SNHG10-Mut promoter (Fig. 7f), validating that ETS1 bound to SNHG10 promoter at the predicted sites. These data indicated that ETS1 activated SNHG10 transcription in glioma.

Further, we probed the function of ETS1 in glioma and whether its regulatory role was mediated by SNHG10/miR-532-3p/FBXL19 pathway. According to the results of colony formation and EdU assays, we viewed that loss of ETS1 evidently restrained cell proliferative ability, while the co-transfection of pcDNA3.1/SNHG10, miR-532-3p inhibitor or pcDNA3.1/FBXL19 abrogated this effect (Additional file 3: Fig. S3A, B). Through conducting caspase 3/8/9 activity detection and TUNEL assay, we certified the facilitative effect of ETS1 knockdown on glioma cell apoptosis, while such impact was then rescued by SNHG10 up-regulation, miR-532-3p repression or FBXL19 overexpression (Additional file 3: Fig. S3C, D). Further, the outcomes of sphere formation assay and RT-qPCR analysis uncovered that the co-transfection of pcDNA3.1/SNHG10, miR-532-3p inhibitor or pcDNA3.1/FBXL19 neutralized the inhibitory influence of ETS1 deficiency on stemness (Additional file 4: Fig. S4A, B). Finally, seen from the results of wound healing and transwell assays, we knew that the hampered migration and invasion of glioma cells with ETS1 deficiency was recovered after further overexpressing SNHG10, inhibiting miR-532-3p or up-regulating FBXL19 (Additional file 4: Fig. S4C, D). Conclusively, ETS1 acted as a transcription activator of SNHG10 and exhibited oncogenic properties via SNHG10/miR-532-3p/FBXL19 axis in glioma.

### Discussion

Glioma is a common tumor among human beings and cannot be well treated with current medical level [22]. Understanding the potential mechanism in glioma may help to find valuable targets for the therapy of glioma.

Mounting proofs have suggested that abnormal expression of lncRNAs could lead to the dysregulation of glioma. As an example, lncRNA H19 promoted the progression of glioma by targeting miR-140/Iaspp axis [23]. Previously, SNHG10 has been reported to exert an oncogenic function in several cancer types like hepatocellular carcinoma (HCC) [24] and gastric cancer (GC) [25]. However, a recent report also argued SNHG10 as a tumor-repressor in non-small cell lung cancer (NSCLC) [26]. In this study, SNHG10 was found to be up-regulated in glioma cells, and it facilitated cell growth, stemness, migration and invasion in glioma, which is consistent with the findings in HCC and GC [24, 27].

Increasing studies have demonstrated that lncRNAs work as miRNA sponges to sequester the combination of miRNAs with mRNAs [28]. In this study, we validated the cytoplasmic localization of SNHG10 in glioma cells, and found miR-532-3p as the downstream molecule of SNHG10. The sponging role of lncRNAs to miR-532-3p has been unveiled in carcinomas such as NSCLC [29]. Interestingly, previous studies have demonstrated that miR-532-3p functioned as a tumor-inhibitor in some malignancies like colorectal cancer [30], but also worked as a tumor-promoter in others like HCC [21]. Here, we revealed that miR-532-3p was down-regulated in glioma cells, and that its overexpression suppressed cell growth and stemness in glioma. Namely, miR-532-3p was a tumor-repressor in glioma, similar to its role in tongue squamous cell carcinoma [20].

Thereafter, we uncovered FBXL19 as the downstream target of miR-532-3p in glioma. Importantly, Xie et al. [31] recognized FBXL19 as one of the core genes in glioblastoma. Our study found that FBXL19 was up-regulated in glioma cells, and down-regulating FBXL19 could restrict the growth and stemness of glioma cells. Although the precise downstream mechanism of FBXL19 in facilitating glioma development was not elucidated here, it functions as a ubiquitin E3 ligase to affect the ubiquitination and degradation of certain proteins has been disclosed by several reports [32, 33]. Next, rescue experiments proved that FOXBL19 was required for the contribution of SNHG10 to gliomagenesis. However, whether there are some other pathways downstream SNHG10 remains covered presently.

Further, we identified ETS1 as the transcriptional activator of SNHG10 in glioma, while the trans-activation role of ETS1 in glioma has been previously described [34]. In addition, ETS1 has been suggested to elicit tumor-facilitating functions in glioma by several former researches [35, 36]. Such phenomenon was also certified by our present work, and we further proved that ETS1 exerted its promotion on glioma development by targeting SNHG10/miR-532-3p/FBXL19 signaling.

However, present work still lacks clinical supports and in-depth animal studies to further validate the importance of SNHG10/miR-532-3p/FBXL19 axis in glioma, and these two aspects will be further focused on in our future research. Also, the downstream mechanism whereby FBXL19 works as a tumor-contributor in glioma, and whether SNHG10 functions in glioma through other pathways, need to be further uncovered in future studies. Moreover, whether targeting SNHG10 will be developed into a new method for treating patients with glioma still needs to be further evidenced in the future.

## Conclusion

Our study found that SNHG10 was an oncogene in glioma and it accelerated the malignant phenotypes of glioma cells by targeting miR-532-3p/FBXL19 axis. Besides, SNHG10 was transcriptionally activated by ETS1. These findings indicated that ETS1/SNHG10/miR-532-3p/FBXL19 axis would be helpful for the exploration of novel targets for glioma treatment.

## Supplementary information

**Additional file 1: Figure S1.** (A, B) The expression of SNHG10 in cells transfected with sh-SNHG10#1/2 and pcDNA3.1/SNHG10 was examined by RT-qPCR. (C) Impact of SNHG10 overexpression on the level of stemness-related genes (OCT4, Nanog and SOX2) was evaluated by RT-qPCR. (D, E) Effect of sh-SNHG10#1/2 or pcDNA3.1/SNHG10 on cell migration was evaluated by wound healing assay. (F, G) The invasive ability of glioma cells transfected with sh-SNHG10#1/2 or pcDNA3.1/SNHG10 was monitored by transwell assay. (H) The levels of miR-2278 and miR-532-3p were detected by RT-qPCR in glioma cells. (I) MiR-532-3p expression was examined by RT-qPCR in cells transfected with NC mimics or miR-532-3p mimics. (J) Cell apoptosis under miR-532-3p upregulation was probed by caspase-8/9 activity detection. **P < 0.01, n.s. presented no significance.

**Additional file 2: Figure S2.** (A) Western blot analyses of FBXL19, NPTN and SRRM2 in glioma cells and normal NHA cells. (B, D) FBXL19 protein level was assessed in cells transfected with miR-532-3p mimics, sh-SNHG10#1/2 or pcDNA3.1/SNHG10. (E) Impact of FBXL19 knockdown on the protein levels of OCT4, Nanog and SOX2 was estimated by western blot. (F, G) Wound healing and transwell assays were carried out to test cell migration and invasion in the groups of sh-NC, sh-SNHG10#1 and sh-SNHG10#1+pcDNA3.1/FBXL19. **P < 0.01.

**Additional file 3: Figure S3.** (A, B) The proliferation of cells transfected with sh-NC, sh-ETS1#1, sh-ETS1#1+pcDNA3.1/SNHG10, sh-ETS1#1+miR-532-3p inhibitor or sh-ETS1#1+pcDNA3.1/FBXL19 was determined by colony formation and EdU assays (scale bar = 100μm). (C, D) Cell apoptosis under above conditions was estimated by caspase activity analysis and TUNEL assay (scale bar = 100μm). **P < 0.01.

**Additional file 4: Figure S4. **(A, B) The stemness of indicated cells was reflected via sphere formation assay and RT-qPCR detection of stemness-related genes. (C, D) Cell migration and invasion capacities under diverse contexts were monitored by wound healing and Transwell assays. **P < 0.01.

## Data Availability

Research data and material have been presented within the present manuscript and the Additional files 1, 2, 3, 4.

## Abbreviations

lncRNAs: Long non-coding RNAs
miRNAs: MicroRNAs
ceRNAs: Competing endogenous RNAs
ChIP: Chromatin immunoprecipitation
RIP: RNA immunoprecipitation
SNHG10: Small nucleolar RNA host gene 10
FBXL19: F-box and leucine rich repeat protein 19
ETS1: ETS Proto-Oncogene 1
