# Supplementary figures and images for: ETS1-activated SNHG10 exerts oncogenic functions in glioma via targeting miR-532-3p/FBXL19 axis
Source: Cancer Cell Int. 2020 Dec 9;20:589. doi: 10.1186/s12935-020-01649-2 (PMC7725120; doi:10.1186/s12935-020-01649-2)

A

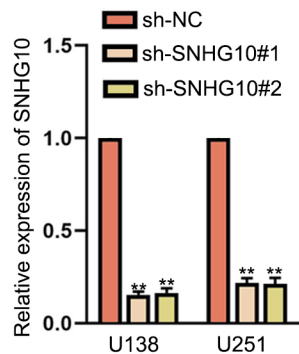

B

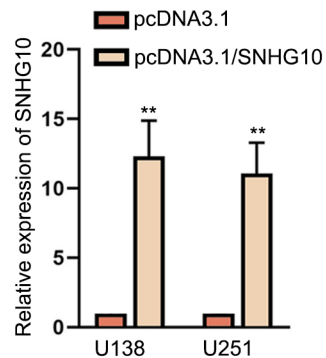

C

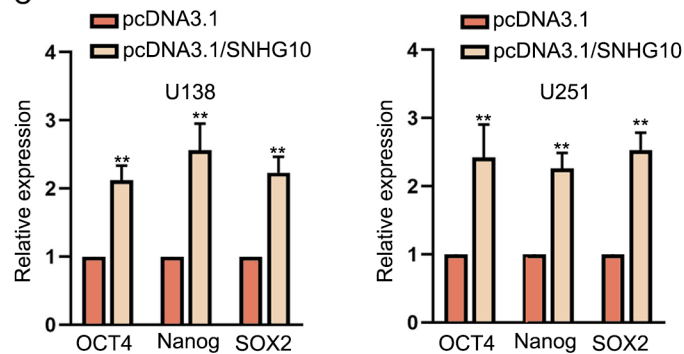

D

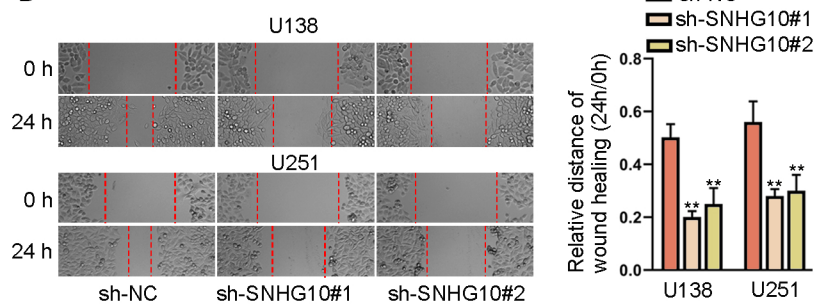

E

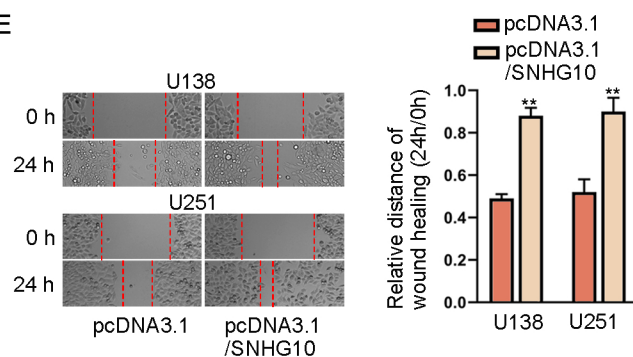

F

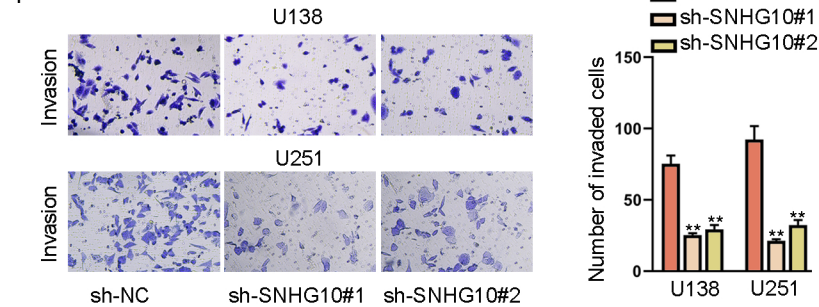

G

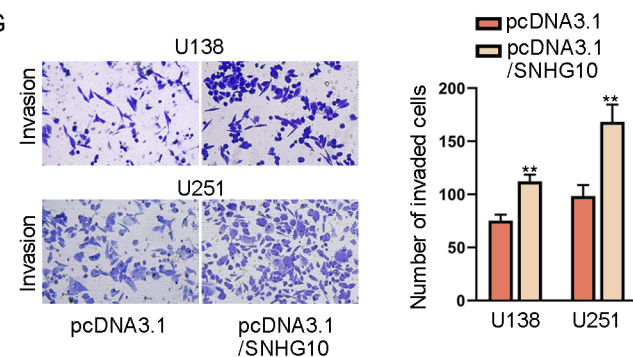

H

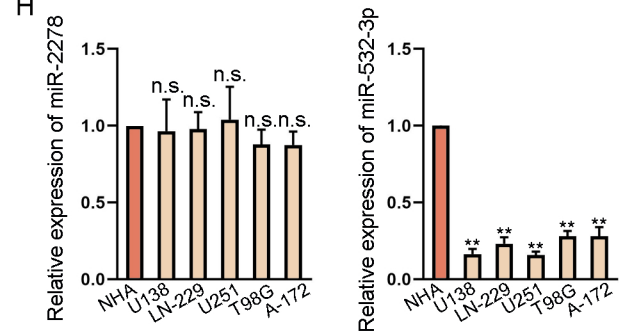

I

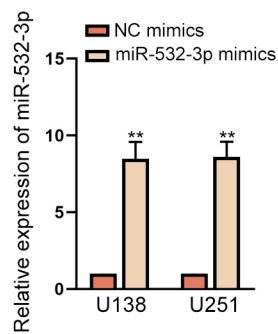

J

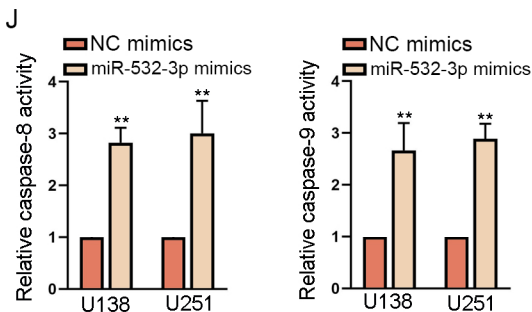

Supplement: Supplementary file 1 — Additional file 1: Figure S1. (A, B) The expression of SNHG10 in cells transfected with sh-SNHG10#1/2 and pcDNA3.1/SNHG10 was examined by RT-qPCR. (C) Impact of SNHG10 overexpression on the level of stemness-related genes (OCT4, Nanog and SOX2) was evaluated by RT-qPCR. (D, E) Effect of sh-SNHG10#1/2 or pcDNA3.1/SNHG10 on cell migration was evaluated by wound healing assay. (F, G) The invasive ability of glioma cells transfected with sh-SNHG10#1/2 or pcDNA3.1/SNHG10 was monitored by transwell assay. (H) The levels of miR-2278 and miR-532-3p were detected by RT-qPCR in glioma cells. (I) MiR-532-3p expression was examined by RT-qPCR in cells transfected with NC mimics or miR-532-3p mimics. (J) Cell apoptosis under miR-532-3p upregulation was probed by caspase-8/9 activity detection. **P < 0.01, n.s. presented no significance. [file 12935_2020_1649_MOESM1_ESM.pdf]

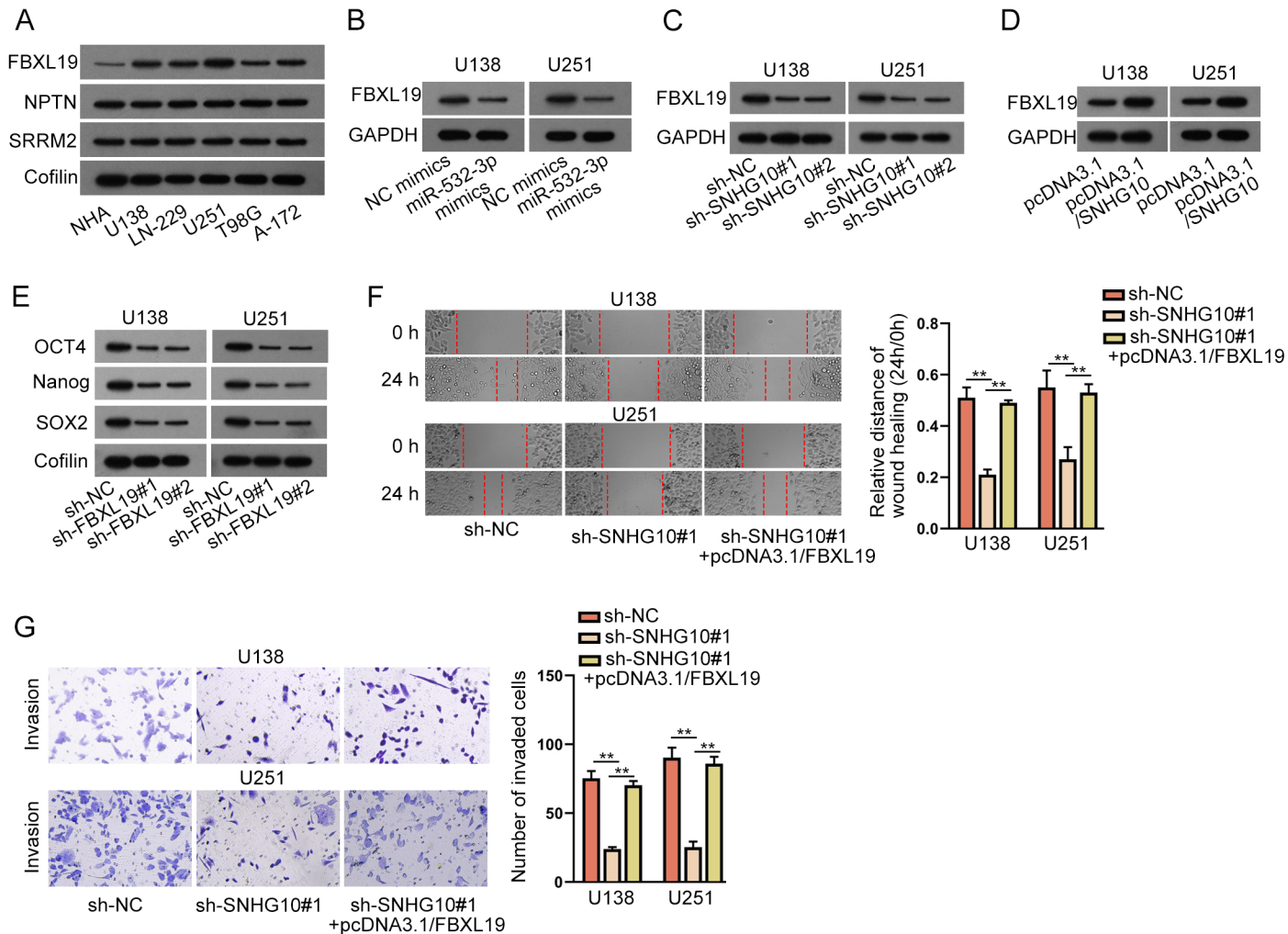

Supplement: Supplementary file 2 — Additional file 2: Figure S2. (A) Western blot analyses of FBXL19, NPTN and SRRM2 in glioma cells and normal NHA cells. (B, D) FBXL19 protein level was assessed in cells transfected with miR-532-3p mimics, sh-SNHG10#1/2 or pcDNA3.1/SNHG10. (E) Impact of FBXL19 knockdown on the protein levels of OCT4, Nanog and SOX2 was estimated by western blot. (F, G) Wound healing and transwell assays were carried out to test cell migration and invasion in the groups of sh-NC, sh-SNHG10#1 and sh-SNHG10#1+pcDNA3.1/FBXL19. **P < 0.01. [file 12935_2020_1649_MOESM2_ESM.pdf]

A

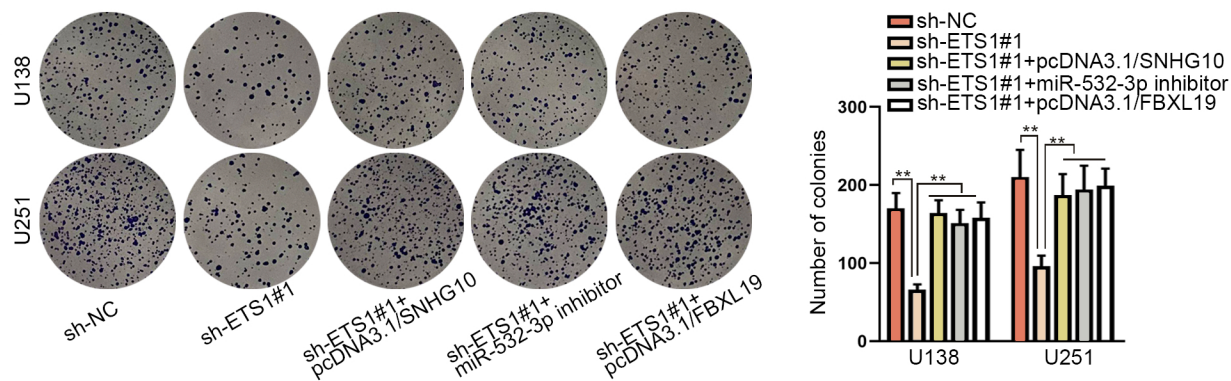

B

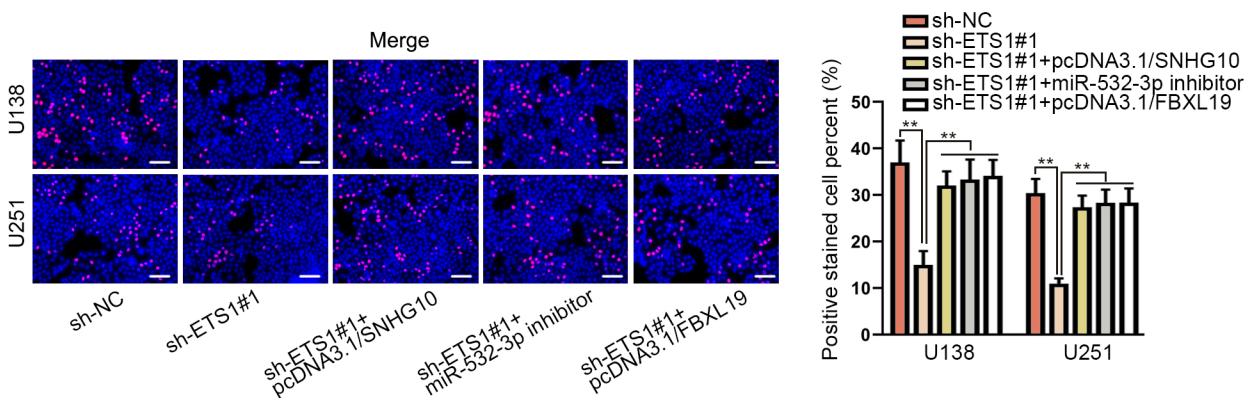

C

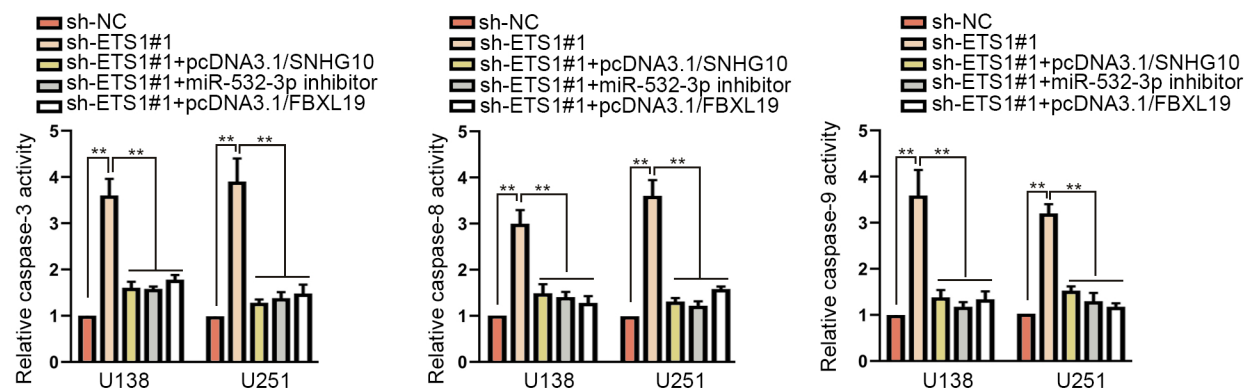

D

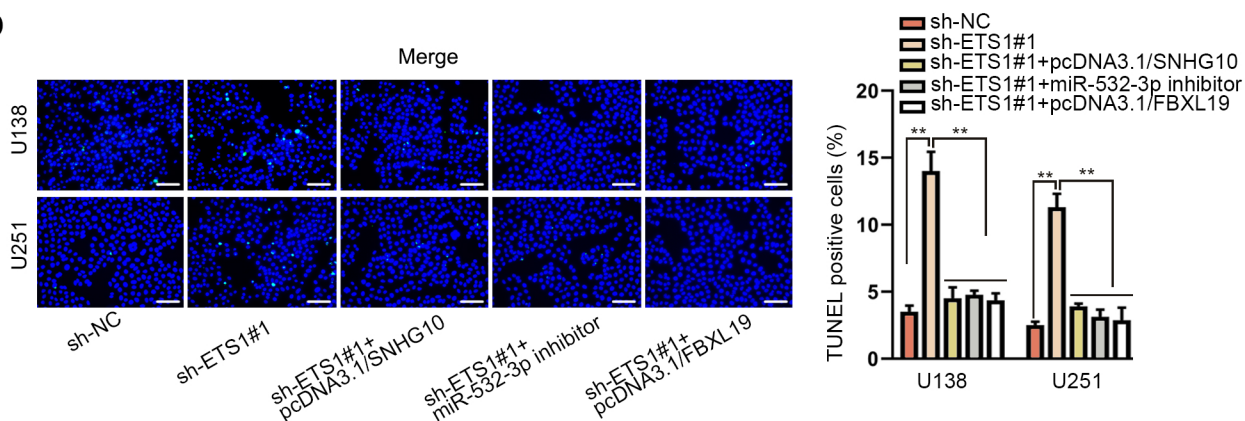

Supplement: Supplementary file 3 — Additional file 3: Figure S3. (A, B) The proliferation of cells transfected with sh-NC, sh-ETS1#1, sh-ETS1#1+pcDNA3.1/SNHG10, sh-ETS1#1+miR-532-3p inhibitor or sh-ETS1#1+pcDNA3.1/FBXL19 was determined by colony formation and EdU assays (scale bar = 100μm). (C, D) Cell apoptosis under above conditions was estimated by caspase activity analysis and TUNEL assay (scale bar = 100μm). **P < 0.01. [file 12935_2020_1649_MOESM3_ESM.pdf]

A

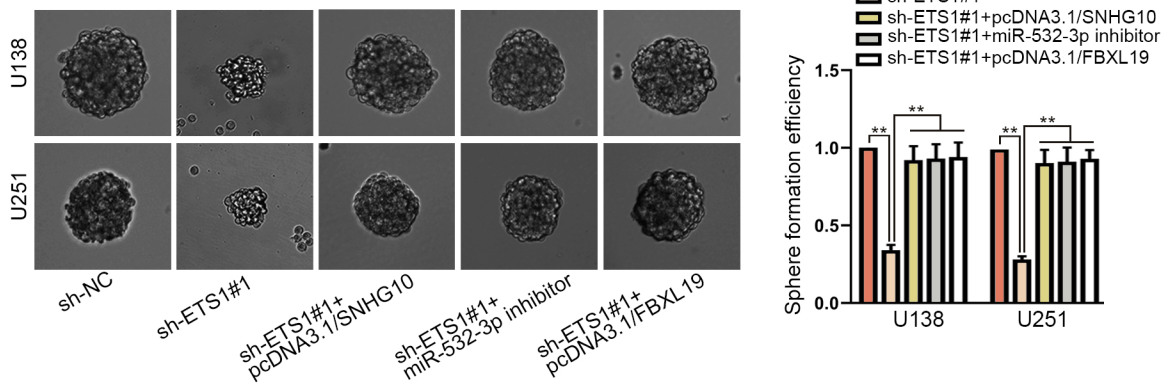

B

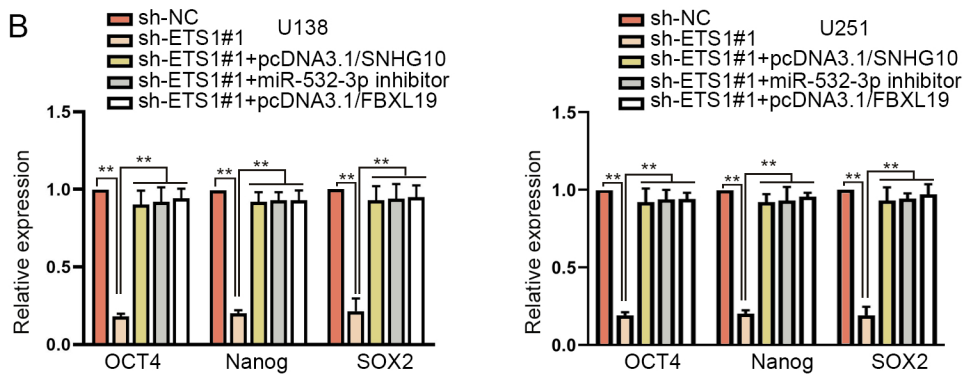

C

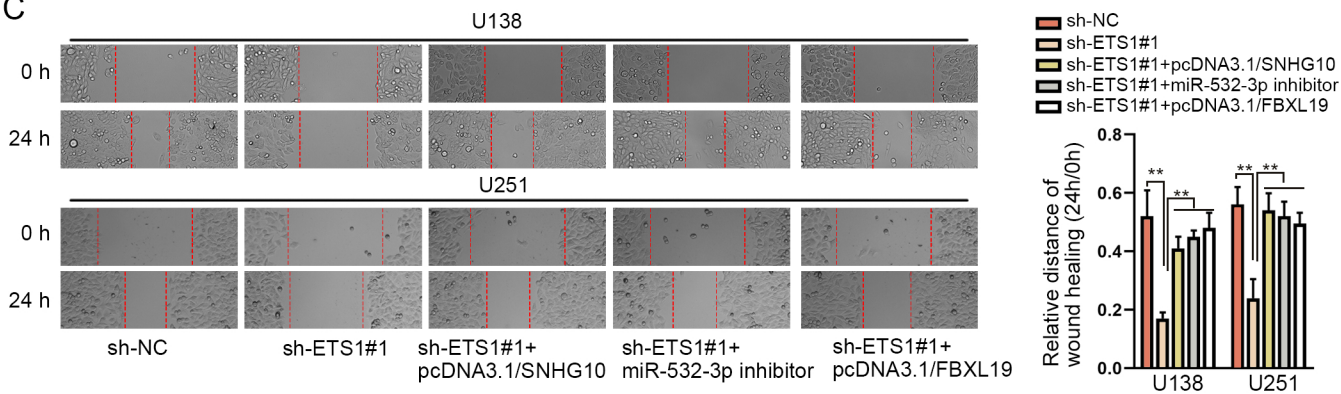

D

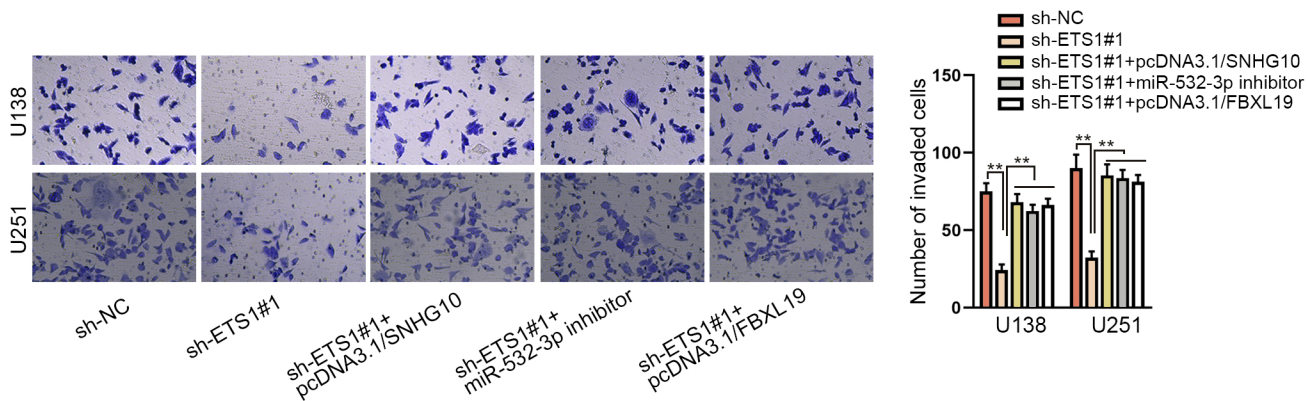

Supplement: Supplementary file 4 — Additional file 4: Figure S4. (A, B) The stemness of indicated cells was reflected via sphere formation assay and RT-qPCR detection of stemness-related genes. (C, D) Cell migration and invasion capacities under diverse contexts were monitored by wound healing and Transwell assays. **P < 0.01. [file 12935_2020_1649_MOESM4_ESM.pdf]
